# Anatomic variation of palmaris longus and flexor digitorum superficialis of little finger in Indian population

**DOI:** 10.1051/sicotj/2015006

**Published:** 2015-06-04

**Authors:** Aakash Mugalur, Sunil M. Shahane, Ashwin Samant, Aditya C. Pathak, Atul Patil, Rajeev Reddy

**Affiliations:** 1 Department of Orthopaedics, R N Cooper Hospital 400056 Vile Parle (W), Mumbai India; 2 Department of Orthopaedics, Terna Medical College 400706 Navi Mumbai India

**Keywords:** Palmaris, Association, Flexor tendon, Variability, Abnormality

## Abstract

*Introduction*: Palmaris longus and flexor digitorum superficialis of the little finger are highly variable anatomically. The tendons vary in different parts of the globe and different regions of the same country. Many studies have negated an association between the tendons. However, most of the studies have a sample size of less than 500 subjects.

*Aims and objectives*: The aim was to study the tendons in the Indian population and study the association, if any between the tendons and to test functional significance of the tendons using the Michigan Hand Outcomes Questionnaire.

*Methods and material*: It was a single centre cross-sectional study with a sample size of 1500 in the age group of 20–60 years. The subjects were tested for the presence of the tendons and their functionality was assessed by Michigan Hand Outcomes Questionnaire. The data was tabulated and was assessed using SPSS 13.0 software.

*Results*: Palmaris longus was bilaterally absent in 10.9% and flexor digitorum superficialis of the little finger was bilaterally absent in 42% of the cases. There was a statistically significant association between the tendons when considered bilaterally. The tendons did not have any bearing on the functionality as assessed by the Michigan Hand Outcomes Questionnaire.

*Conclusions*: There is significant variability in the palmaris longus and flexor digitorum superficialis tendon to the little finger not only in the different countries but in different regions of the same country. However despite the various clinical and medicolegal aspects concerning both the tendons, they do not have much bearing on the functionality of the hand.

## Introduction

Palmaris longus is a muscle of the superficial flexor compartment of the forearm. It inserts into the distal half of the flexor retinaculum and at the apex of the palmar aponeurosis. It is supplied by the median nerve and acts as a tensor of the palmar aponeurosis apart from acting as a weak flexor of the wrist. This phylogenetically degenerate expendable tendon assumes importance because of its anatomical variability, wide variation in the reported prevalence of absence with respect to different ethnic groups [12, 18, 26] and its use as a tendon graft in various reconstructive surgeries of upper limb, lip augmentation, ptosis correction and facial palsy management [2, 6, 11, 14]. Its phylogenicity is suggested by a short muscle belly, a long tendon and replacement of a distal tendon by the ligamentous palmar aponeurosis. A study by Wehbe concluded that absence appears to be hereditary but genetic transmission was not clear [25]. Palmaris longus agenesis differs according to race, sex, and to the right and left sides. There is a wide variation in the incidence of palmaris longus ranging from 0% to 63% with an overall 16% unilateral and 9% bilateral absence described in the literature [4, 16].

Absence of flexor digitorum superficialis function in the little finger is a relatively common congenital anomaly that can complicate assessment of little finger tendon injuries. Absence of little finger superficialis function in one hand was not found to be a reliable indicator of this function in the opposite hand [24]. Various studies have negated any relationship between absent palmaris longus and flexor digitorum superficialis of the little finger [10, 20, 23]. In the Indian setup a study by Sharma et al. [21] had similar conclusions with respect to the association between the tendons. A study by Agarwal et al. [1] concluded that the prevalence of the unilateral absence of the palmaris longus tendon in an Indian population is comparable to the Western population but a bilateral absence is significantly less. Differing from other studies Agarwal et al. concluded that in patients with an absent palmaris longus tendon, the flexor digitorum superficialis of the little finger is weak, especially in males.

The aims and objectives of our study were: (1) To study the absence of palmaris longus and flexor digitorum superficialis of the little finger in the Indian population. (2) To study the association between absent palmaris longus and weak or absent flexor digitorum superficialis of the little finger. (3) To study the functional significance of palmaris longus and flexor digitorum superficialis of little finger using the Michigan Hand Outcomes Questionnaire.

## Materials and methodology

The study was a cross-sectional study conducted at our institute after obtaining clearance from the Institutional Ethics Committee. All the participants gave formal consent before enrolling for the study. We included individuals between the age group of 20 and 60 years for the ease of explaining the various methods of testing to the participants. The exclusion criteria included upper limb trauma patients in whom the bilaterality cannot be studied effectively, persons who have undergone previous tendon transfer operations in the upper limb, patients with nerve injuries and wrist and hand deformities. Any patient with hemiparesis, monoparesis or quadriparesis and those subjects who were unwilling to take part in the study were excluded.

**Figure 1.**
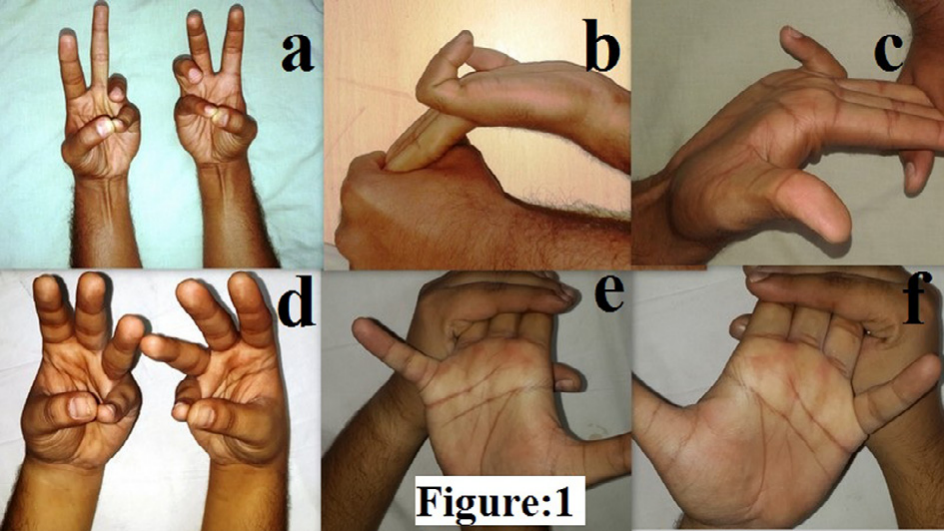
(a) Schaeffer’s test demonstrating the presence of palmaris longus bilaterally, (b) and (c) standard test demonstrating the functional presence of flexor digitorum superficialis of the little finger bilaterally, (d) Schaeffer’s test demonstrating the absence of palmaris longus bilaterally, (e) and (f) standard test demonstrating the functional absence of flexor digitorum superficialis of the little finger bilaterally.

We used a battery of tests for the palmaris longus tendon. Palmaris longus tendon was declared as absent when all the tests were negative and was considered as present with a single test being positive for the tendon. We primarily used Schaeffer’s [19] test for our subjects – opposition of the thumb and the little finger with flexion of the wrist leads to the prominence of the palmaris longus tendon at the wrist (Figure 1). In cases of ambiguity we used Mishra’s Test I & II [13] and Pushpakumar et al.’s two-finger sign’ test [17]. We used the following tests before we declared palmaris longus as absent: Thompson et al.’s test [22], Gangata test [8], Oudit et al.’s four-finger sign [15], Lotus test and Bhattacharya et al.’s test [3]. The presence of the flexor digitorum superficialis of the little finger was tested by asking the patient to flex the little finger with the rest of the fingers positioned in extension at the inter-phalangeal joints to negate the action of flexor digitorum profundus (Figure 1). A modification of the standard test wherein the fourth and the fifth digit were allowed to flex together was the second test used. The flexor digitorum superficialis was declared absent in case of inability to flex the little finger within 20° of the passive range of motion of the finger [9]. The hand function was evaluated using the “Michigan Hand Outcomes Questionnaire” [5] to evaluate the functional implication of the palmaris longus tendon and the flexor digitorum superficialis tendon to the little finger.

## Results

Subjects (1500) participated in the study which included 557 females and 943 males. The mean age was 34.66 years. Palmaris longus was bilaterally absent in 10.9% of the cases. Unilateral absence on the right side was 2.7% and was 5.7% on the left. Flexor digitorum superficialis of the little finger was bilaterally absent in 42% of the cases. Unilateral absence on the right side was 6.1% and was 8.6% on the left. There was a statistically significant association between the palmaris longus and the flexor digitorum superficialis tendons on the right side (Table 1), however the association was not significant on the left side (Table 2). When the tendons are considered bilaterally there is a statistically significant association between the palmaris longus and FDS tendon to the little finger (Table 3). Right side palmaris longus is a significant predictor of right side-FDS of little finger; however, left side palmaris longus is not a significant predictor of left side-FDS of little finger. However, the presence or absence of the tendons had no bearing on the functionality assessed by the Michigan Hand Outcomes Questionnaire.

**Table 1.** Association among the cases between right side-palmaris longus and right side-FDS* of little finger.

Right side-Palmaris longus		Right side-FDS of little finger		Total
		Present	Absent	
Present	No.	690	607	1297
	%	53.2%	46.8%	100.0%
Absent	No.	89	114	203
	%	43.8%	56.2%	100.0%
Total	No.	779	721	1500
	%	51.9%	48.1%	100.0%

Chi-square tests	Value	*df*	*p*-value	Association is-

Pearson Chi-square	6.157	1	0.013	Significant
Continuity correction	5.788	1	0.016	Significant

Significant association between right side palmaris longus and right side flexor digitorum superficialis* of the little finger. *p*-value = .013 (<.05).

**Table 2.** Association among the cases between left side-palmaris longus and left side-FDS* of little finger.

Left side-Palmaris longus		Left side-FDS of little finger		Total
		Present	Absent	
Present	No.	623	628	1251
	%	49.8%	50.2%	100.0%
Absent	No.	118	131	249
	%	47.4%	52.6%	100.0%
Total	No.	741	759	1500
	%	49.4%	50.6%	100.0%

Chi-square tests	Value	*df*	*p*-value	Association is-

Pearson Chi-square	0.483	1	0.487	Not significant
Continuity correction	0.391	1	0.532	Not significant

Insignificant association between left side palmaris longus and left side flexor digitorum superficialis* of the little finger. *p*-value = .487 (>.05).

**Table 3.** Association among the cases between palmaris longus and FDS of little finger when considered bilaterally.

Palmaris longus		FDS of little finger		Total
		Present	Absent	
Present	No.	529	683	1212
	%	43.6%	56.4%	100.0%
Absent	No.	83	76	159
	%	52.2%	47.8%	100.0%
Total	No.	612	759	1371
	%	44.6%	55.4%	100.0%

Chi-square tests	Value	*df*	*p*-value	Association is-

Pearson Chi-square	4.162	1	0.0413	Significant
Continuity correction	3.823	1	0.0505	Not significant

Significant association between palmaris longus and flexor digitorum superficialis* of the little finger when considered bilaterally. *p*-value = .0413 (<.05).

## Discussion

Palmaris longus is a muscle of the superficial compartment of the forearm. It is a phylogenetically degenerate retrogressive muscle; it is one of the most variable muscles in humans, showing variation in position, duplication and slips [10]. Palmaris longus has evolved and it differs in various mammalian taxa with respect to the nerve supply, its origin and its derivation from the parent muscle [7] and is an active muscle in arboreal primates used for prehensile progression from tree to tree. Although phylogenetically degenerate, palmaris longus tendon with the advantage of expendability, is one of the most sought-after tendons for tendon transfer by the reconstructive surgeons and has proved to be of use in various reconstructive procedures [2, 6, 11, 14].

The agenesis of palmaris longus is variable in different parts of the world and surprisingly in different parts of the same country [1, 10, 19–21, 23]. Congenital absence of the flexor digitorum superficialis of the little finger is a relatively common congenital anomaly and could be coexistent with an absent palmaris longus. However most of the studies negated a statistically significant association between the two tendons [1, 10, 20, 21, 23]. We studied the prevalence of the absence of both the tendons and whether they were associated with each other.

In our study the Palmaris longus was bilaterally absent in 10.9% of the cases. This is higher as compared to some of the Indian studies [1]. Unilateral absence on the right side was 2.7% and was 5.7% on the left side. The overall absence was 19.3%, which was higher when compared to Chinese, Korean population but was lower compared to that of Caucasian, Nigerian, Irish and Turkish population [4, 10, 20, 23, 25]. In our study the overall absence of flexor digitorum superficialis to the little finger was significantly highly when compared to the other studies [1, 10, 23]. Flexor digitorum superficialis of the little finger was bilaterally absent in 42% of the cases. Unilateral absence on the right side was 6.1% and was 8.6% on the left.

Our study suggested that there could be a statistically significant association between the palmaris longus and the flexor digitorum superficialis tendons on the right side, however the association was not significant on the left side. When the tendons are considered bilaterally there could be a statistically significant association between the palmaris longus and FDS tendon to the little finger (*p* = 0.0413 with an error of .05). This result is not comparable to most of the studies in the literature which rule out any statistically significant association between the tendons [1, 10, 20, 21, 23]. However, the error of .05 of the value of *p* should be considered as it could be a false positive association.

We also analysed the functionality of the hands in various subjects using the Michigan Hand Outcomes Questionnaire. There was no difference with respect to functionality and it was independent of the presence or absence of the tendons in question.

The presence or absence of palmaris long FDS assumes importance in view of tendon transfers. The absence of FDS may create ambiguity in the acute trauma setting during evaluation of little finger lacerations [24]. It may have a bearing on medicolegal importance in the Indian setup where any tendon injury is considered as grievous hurt. The palmaris longus tendon apart from the aforementioned uses in transfers and reconstructive surgery is also of some clinical importance. Apart from protecting the median nerve from trauma, its anatomical location is used as a guide in corticosteroid injections in cases of carpal tunnel syndrome and median nerve block in anaesthesia.

In conclusion there is significant variability in the palmaris longus and flexor digitorum superficialis tendon to the little finger not only in the different countries but in different regions of the same country. However despite the various clinical and medicolegal aspects concerning both the tendons, they do not have much bearing on the functionality of the hand.

## Conflicts of interest

The authors (Dr. Aakash Mugalur, Dr. Sunil M Shahane, Dr. Ashwin Samant, Dr. Aditya C Pathak, Dr. Atul Patil, Dr. Rajeev Reddy), their immediate families, and any research foundation with which they are affiliated have not received any financial payments or other benefits from any commercial entity related to the subject of this article. Further there is no association with any organization commercial or otherwise which might pose a conflict of interest with respect to the submitted article.
